# Fatty Acid Metabolism Provides an Essential Survival Signal in OxPhos and BCR DLBCL Cells

**DOI:** 10.3390/biomedicines13030707

**Published:** 2025-03-13

**Authors:** Aurélie Montagne, Konstantina Kotta, Karoline Kielbassa-Elkadi, Isabelle Martins, José Ángel Martinez-Climent, Guido Kroemer, Catherine Thieblemont, Véronique Baud

**Affiliations:** 1 NF-κB, Differentiation and Cancer, Université Paris Cité, 75006 Paris, France; aurelie.montagne@outlook.fr (A.M.); kn.kotta@gmail.com (K.K.); karoline.el-kadi@u-paris.fr (K.K.-E.); catherine.thieblemont@aphp.fr (C.T.); 2 Equipe Labellisée Ligue contre le Cancer, Cordeliers Research Center, INSERM U1138, Université Paris Cité, Sorbonne Université, 75006 Paris, France; isabellemart@gmail.com (I.M.); kroemer@orange.fr (G.K.); 3 Metabolomics and Cell Biology Platforms, Gustave Roussy Comprehensive Cancer Institute, 94800 Villejuif, France; 4 Department of Hematology, Center for Applied Medical Research, University of Navarra, IDISNA, CIBERONC, 31071 Pamplona, Spain; jamcliment@unav.es; 5 Institut du Cancer Paris CARPEM, Department of Biology, Hôpital Européen Georges Pompidou, AP-HP, 75015 Paris, France; 6 Hemato-Oncology, AP-HP, Hôpital Saint-Louis, Université Paris Cité, 75006 Paris, France

**Keywords:** B-cell lymphoma, DLBCL, survival, metabolism, fatty acid, mitochondrial stress

## Abstract

**Backgroung/objectives:** Diffuse large B-cell lymphoma (DLBCL) is the most frequent subtype of malignant lymphoma and is a heterogeneous disease with various gene and chromosomal abnormalities. The development of novel therapeutic treatments has improved DLBCL prognosis, but patients with early relapse or refractory disease have a poor outcome (with a mortality of around 40%). Metabolic reprogramming is a hallmark of cancer cells. Fatty acid (FA) metabolism is frequently altered in cancer cells and recently emerged as a critical survival path for cancer cell survival. **Methods:** We first performed the metabolic characterization of an extended panel of DLBCL cell lines, including lipid droplet content. Then, we investigated the effect of drugs targeting FA metabolism on DLBCL cell survival. Further, we studied how the combination of drugs targeting FA and either mitochondrial metabolism or mTOR pathway impacts on DLBCL cell death. **Results:** Here, we reveal, using a large panel of DLBCL cell lines characterized by their metabolic status, that targeting of FA metabolism induces massive DLBCL cell death regardless of their OxPhos or BCR/glycolytic subtype. Further, FA drives resistance of DLBCL cell death induced by mitochondrial stress upon treatment with either metformin or L-asparaginase, two FDA-approved antimetabolic drugs. Interestingly, combining inhibition of FA metabolism with that of the mTOR oncogenic pathway strongly potentiates DLBCL cell death. **Conclusion:** Altogether, our data highlight the central role played by FA metabolism in DLBCL cell survival, independently of their metabolic subtype, and provide the framework for the use of drugs targeting this metabolic vulnerability to overcome resistance in DLBCL patients.

## 1. Introduction

Diffuse large B-cell lymphoma (DLBCL) is the most common type of B-cell non-Hodgkin’s lymphoma (NHL), accounting for around 40% of all NHLs worldwide [1,2]. Although standard immunochemotherapy R-CHOP (rituximab, cyclophosphamide, doxorubicin, vincristine, and prednisone) achieves a cure rate of about 60% of DLBCL patients, 40% of DLBCL patients experience relapse, remain refractory to conventional treatment, progress, and die [1,3]. Recently, cutting-edge immunotherapies by CAR-T cells have improved outcomes of refractory/relapsed DLBCL patients, but 60% will still succumb [4,5,6]. Therefore, it is necessary to explore novel vulnerabilities and therapeutic strategies for patients with DLBCL. DLBCL is a highly heterogeneous disease with various gene and chromosomal abnormalities and oncogenic drivers [7,8,9,10]. DLBCL can be classified into several molecular subtypes identified through coordinated expression of gene clusters [11,12]. The cell of origin (COO) classification has uncovered two molecular subtypes: germinal center B-cell-like (GCB) and activated B-cell-like (ABC) DLBCLs [11]. More recently, metabolic signatures uncovered distinct metabolic DLBCL subsets [12], including the OxPhos DLBCL subset that is characterized by a non-functional BCR and an increased mitochondrial metabolism that is required for this DLBCL subset survival, and the BCR subset that presents an upregulation of genes encoding BCR signaling components and enzymes associated with glycolysis [12,13,14].

Cancer cells exhibit bioenergetic alterations in order to meet their high-energy requirement, and rewiring of cancer cell metabolism is now recognized as a hallmark of cancer [15]. Therefore, targeting tumor energy metabolism has opened up a potential therapeutic window. High glucose consumption through aerobic glycolysis is well known as the “Warburg effect”, but recent discoveries have shown that amino acid as well as lipid and fatty acid (FA) metabolism are also critical for tumorigenesis [16,17,18,19]. However, how metabolism reprogramming, especially FA metabolism, impacts DLBCL cell survival and contributes to poor DLBCL patient outcome is still an emerging field.

Fatty acids (FA) are the main building blocks of several lipid species, including phospholipids, sphingolipids, and triglycerides and are a major source of energy for cancer cell survival via FA β-oxidation (FAO) [17,18]. Cancer cells are constantly replenished with FA via three main routes that consist of uptake of exogenous FA, de novo FA synthesis, and lipolysis from lipid droplets [18,20,21,22]. How intracellular FA metabolism affects DLBCL cell survival and whether the metabolic status of DLBCL cells impacts FA-dependent survival remains poorly defined.

In the study presented here, we demonstrate for the first time in a large panel of DLBCL cell lines characterized by their metabolic status that targeting FAO and FA neosynthesis induces massive DLBCL cell death, both in the OxPhos and BCR/glycolytic subtypes. Further, FA drives resistance to DLBCL cell death induced by mitochondrial stress upon treatment with either metformin or L-asparaginase, two FDA-approved antimetabolic drugs. Interestingly, combining inhibition of FA metabolism with the mTOR oncogenic pathway strongly potentiates DLBCL cell death. Altogether our data highlight the central role played by FA metabolism in DLBCL cell survival, independently of their metabolic subtype, and provide the framework for the use of drugs targeting this metabolic vulnerability to overcome resistance in DLBCL patients.

## 2. Materials and Methods

### 2.1. Human DLBCL Cell Lines and Culture Conditions

ABC DLBCL cell lines (MD901, OCI-Ly3) and GCB DLBCL cell lines (Karpas 422 (K422), OCI-Ly1, OCI-Ly19, OCI-Ly7, OCI-Ly8, SU-DHL4, SU-DHL5, SU-DHL6, SU-DHL10) were obtained from José Angel Martinez-Climent (Centro de Investigación Médica Aplicada, Pamplona, Spain). All DLBCL cell lines were cultured in RPMI-1640/GlutaMAX medium (Gibco, Life Technologies Limited, Paisley, UK) supplemented with 10% heat-inactivated fetal bovine serum (FBS) (Corning, Glendale, AZ, USA), 100 U/mL penicillin, and 100 µg/mL streptomycin (Gibco, Life Technologies Limited, Paisley, UK) in an incubator at 37 °C with 5% CO_2_.

### 2.2. Reagents

Metformin (PHR1084), 2-deoxy-D-glucose (2-DG, D8375), rapamycin (553211), C75 (C5490), and TVB-3166 (SML1694) were purchased from Merck (Darmstadt, Germany), 4-BrCA (A698521) from AmBeed (Arlington Hts, IL, USA), and L-asparaginase (^®^ Kidrolase) was a kind gift from Isabelle Madelaine-Chambrin and Nathalie Jourdan (Service de Pharmacie Centrale, Hôpital Saint-Louis, Paris, France).

### 2.3. Measurement of Intracellular ATP Content

Intracellular ATP content was measured using an ATP luminescence detection kit (CellTiter-Glo^®^ Luminescent Cell Viability Assay, Promega, Madison, WI, USA) according to the manufacturer’s instructions. Briefly, 4 × 10^5^ cells were seeded in 100 µL of complete medium. Cells were treated for 1 h with or without 20 mM 2-deoxy-D-glucose (2-DG) at 37 °C followed by 30 min at room temperature to inhibit glycolysis. At the end of the incubation time, 100 µL of CellTiter-Glo solution was added, and cells were incubated for 10 min at room temperature. The luminescence signal (relative luminescence units (RLU)) was recorded with a microplate reader (Centro LB 960 microplate luminometer, Berthold Technologies, Bad Wildbach, Germany). The difference between total ATP content and ATP produced under 2-DG treatment results in glycolytic ATP.

### 2.4. Apoptosis Assays

DLBCL cells were harvested and washed twice with cold PBS. Cells were resuspended in 1X binding buffer containing Annexin V/PE (BD Biosciences Pharmingen, San Diego, CA, USA) and 4′,6-diaminidino-2-phenylindole (DAPI, Molecular Probes, Life Technologies, Carlsbad, CA, USA) for 15 min at room temperature. The samples were subjected to cytometric analysis with a MACSQuant Analyzer 10 Flow Cytometer (Miltenyi Biotec, Bergisch Gladbach, Germany) and the data were analyzed using the Flowjo v10.2 software (Ashland, OR, USA).

### 2.5. Cell Viability Assay

The same number of cells (1.0 × 10^5^ cells/mL) were cultured in 12-well plates for the indicated periods of time (24 h and 48 h). Cell viability was then assessed using the trypan blue dye exclusion with stained (non-viable) and unstained (viable) cells.

### 2.6. Lipid Droplet Content

Cells were stained with 2 µM BODIPY 493/503 (Invitrogen, Life Technologies Limited, Paisley, UK, D3922) for 30 min at 37 °C in PBS. Then, cells were stained with 1 µg/mL of DAPI for 5 min at room temperature to evaluate lipid droplet content in living cells. The samples were subjected to cytometric analysis with a MACSQuant Analyzer 10 Flow Cytometer (Miltenyi Biotec, Bergisch Gladbach, Germany) and the data were analyzed using the Flowjo v10.2 software (Ashland, OR, USA).

### 2.7. Statistical Analysis

Statistical significance was assessed using an unpaired Student’s *t*-test or one-way ANOVA test (Prism 8.0, GraphPad Software, San Diego, CA, USA). A *p*-value of 0.05 was considered statistically significant with the following degrees: * *p* < 0.05; ** *p* < 0.01; *** *p* < 0.001; **** *p* < 0.0001.

## 3. Results

### 3.1. Metabolic Characterization of an Extended Panel of DLBCL Cell Lines

**Figure 1:**
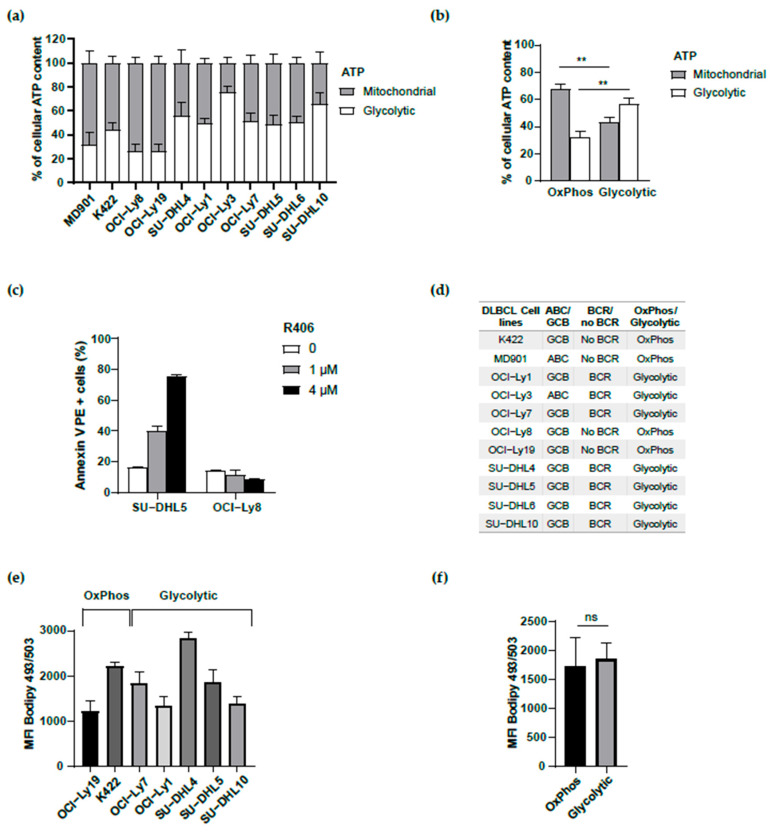
Metabolic characterization of an extended panel of DLBCL cell lines. (**a**) Intracellular levels of glycolytic and mitochondrial ATP of the extended DLBCL cell lines panel. A rate above 50% of glycolytic ATP defined a glycolytic cell line. Error bars represent means ± SD (*n* = 4 individual experiments, each performed in duplicates). (**b**) Percent contribution of glycolysis and mitochondrial metabolism to total cellular ATP. For each subtype, cumulative data from the four OxPhos DLBCL (MD901, K422, OCI-Ly8, OCI-Ly19) and the seven BCR DLBCL (SU-DHL4, OCI-Ly1, OCI-Ly3, OCI-Ly7, SU-DHL5, SU-DHL6, SU-DHL10) cell lines characterized in (**a**) are shown. Error bars represent means ± SEM, *p* values by unpaired Student’s *t*-test, ** *p* < 0.01. (**c**) Apoptosis upon R406 defined OCI-Ly8 as an OxPhos and SU-DHL5 as a BCR DLBCL cell line. OCI-Ly8 and SU-DHL5 DLBCL cell lines were incubated with the indicated concentration of the SYK inhibitor R406 for 96 h (except SU-DHL5 for 24h because of its very high sensitivity to R406), and apoptosis was monitored by Annexin V-PE and DAPI staining followed by FACS analysis. Data are presented as mean values ± SD (*n* = 3). (**d**) Summary of the metabolic (OxPhos vs. glycolytic), BCR, and COO characteristics of the extended panel of DLBCL cell lines. (**e**) Lipid droplet content of DLBCL cell lines by BODIPY 493/503 staining. Data are presented as mean values ± SD, at least *n* = 2 experiments. MFI: median fluorescence intensity. (**f**) Cumulative data from lipid droplet content of OxPhos DLBCL cell lines (OCI-Ly19, K422) versus glycolytic DLBCL cell lines (OCI-Ly7, SU-DHL4, OCI-Ly1, SU-DHL5, SU-DHL10). Data are presented as mean values ± SEM, *p* values by unpaired Student’s *t*-test. ns: not significant.

To explore the contribution of fatty acid metabolism to DLBCL cell survival and its possible relationship with their metabolic and/or BCR status, it was central to use a large panel of DLBCL cell lines whose metabolic status was defined. We used a large panel of eleven different DLBCL cell lines (provided by J-A Martinez-Climent), namely K422, MD901, OCI-Ly1, OCI-Ly3, OCI-Ly7, OCI-Ly8, OCI-Ly19, SU-DHL4, SU-DHL5, SU-DHL6, and SU-DHL10. However, since the metabolic status of several of them was still unknown, we first established whether these DLBCL cell lines were OxPhos or glycolytic DLBCL cell lines. We thus evaluated the level of production of glycolytic ATP in all eleven DLBCL cell lines. Four DLBCL cell lines exhibited a low level of glycolytic ATP (MD901, K422, OCI-Ly8, and OCI-Ly19) compared to the seven other DLBCL cell lines (OCI-Ly1, OCI-Ly3, OCI-Ly7, SU-DHL4, SU-DHL5, SU-DHL6, and SU-DHL10) (Figure 1a,b). R406 is an inhibitor of the spleen tyrosine kinase (SYK) that only induces apoptosis in DLBCL cell lines having an active BCR signaling, whereas non-BCR DLBCL cell lines that do not display functional BCR signaling are insensitive to this inhibitor. Two (SU-DHL5 and OCI-Ly8) out of our eleven DLBCL cell lines have never been evaluated for their R406 sensitivity. As presented in Figure 1c, OCI-Ly8 was insensitive to R406, whereas SU-DHL5 was highly sensitive to R406 at the same doses. Altogether, our data allowed us to classify the large panel of eleven DLBCL cell lines for their metabolic OxPhos vs. glycolytic status as well as for their BCR vs. non-BCR status (Figure 1d). Further, since lipid droplets are now well recognized as an important source of fatty acids (FAs) in cancer cells [20,21], we assessed a potential difference in lipid droplet content in OxPhos vs. glycolytic DLBCL cell lines. Interestingly, no significant differences were observed in the lipid droplet content between OxPhos and glycolytic DLBCL cell lines (Figure 1e,f).

### 3.2. Inhibition of Fatty Acid Oxidation (FAO) Induces Massive DLBCL Cell Death Both in ABC and GCB, OxPhos, and BCR/Glycolytic Subtypes

**Figure 2:**
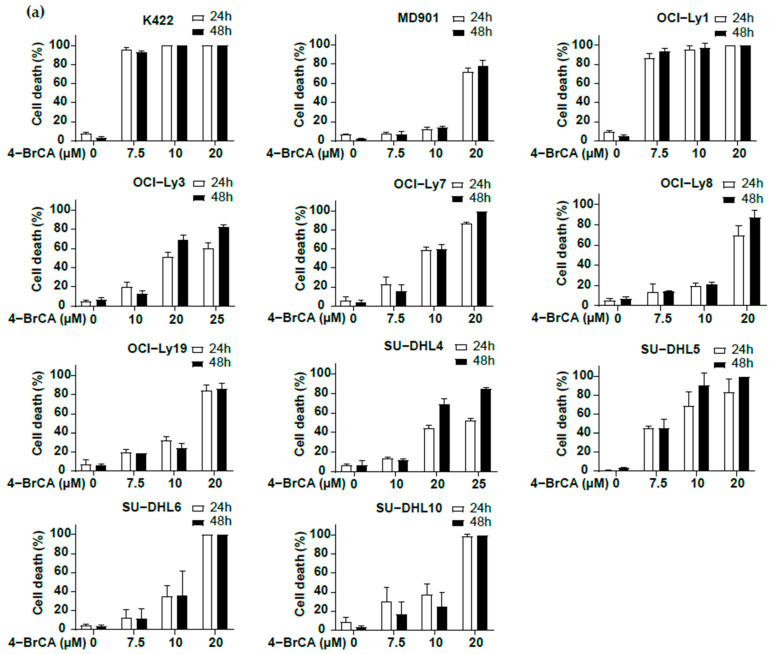

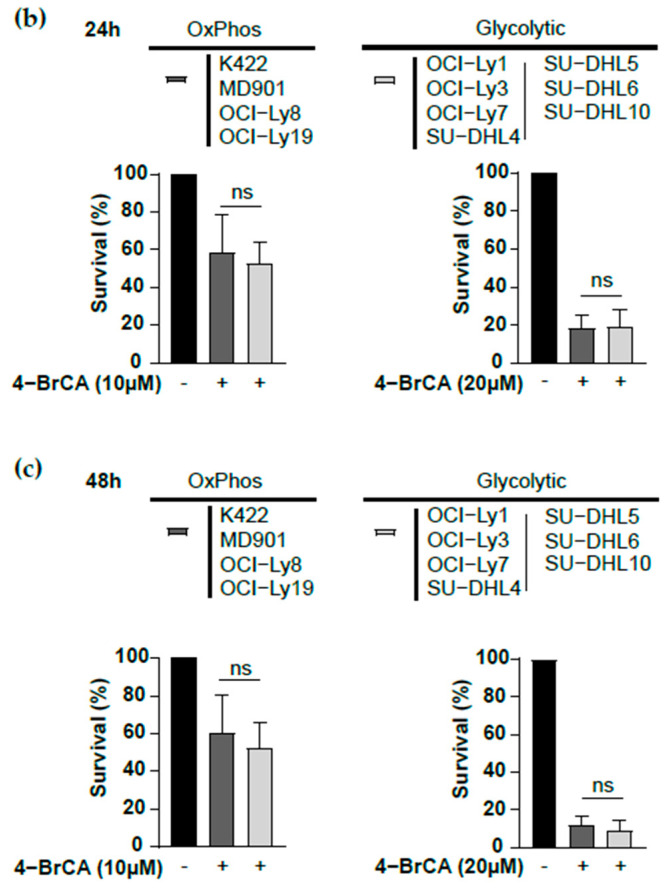
Inhibition of fatty acid oxidation (FAO) induces massive DLBCL cell death both in OxPhos and glycolytic cell lines. (**a**) Inhibition of FAO by 4-BrCA induces massive cell death in DLBCL cell lines. The indicated DLBCL cell lines were treated with 4-BrCA at 7.5, 10, 20, 25 µM for 24 h and 48 h, and monitored for cell death by trypan blue dye exclusion. Error bars represent means ± SD, at least *n* = 2 experiments. (**b**,**c**) DLBCL cell death induced by FAO inhibition is independent of the OxPhos or glycolytic metabolic status. Cell survival relative to untreated cells using 4-BrCA at 10 μM (left) and 20 μM (right) for 24 h (**b**) and 48 h. (**c**) Control condition was set at 100%. Error bars represent means ± SEM, *p* values by unpaired Student’s *t*-test, ns: not significant. Dark grey columns represent OxPhos DLBCL cell lines and light grey columns represent glycolytic DLBCL cell lines.

While it is reported that exogenous palmitate increases mitochondrial respiration and survival of OxPhos DLBCL cells [14], whether basal FAO provides (i.e., in the absence of high levels of exogenous FA) an important survival signal in OxPhos and BCR/glycolytic DLBCL cells is currently unknown. We thus evaluated the impact of 4-bromocrotonic acid (4-BrCA) that irreversibly inhibits FAO of long-, medium-, and short-chain FAs [23] on OxPhos and glycolytic DLBCL cell lines. Strikingly, 4-BrCA induced a marked dose-dependent induction (from 7.5 to 20 µM) of DLBCL cell death in most tested DLBCL cell lines (Figure 2a). The two OxPhos/GCB K422 and glycolytic/GCB OCI-Ly1 DLBCL cell lines were particularly sensitive, with almost all DLBCL cells being killed at 7.5 µM after a treatment of only 24 h (Figure 2a). Interestingly, no significant differences in DLBCL cell survival upon FAO inhibition were observed between OxPhos and glycolytic DLBCL cell lines (Figure 2b,c). Altogether, these data indicate that blocking FAO induces massive DLBCL cell death independently of current metabolic classification.

### 3.3. Inhibition of FASN Induces DLBCL Cell Death

**Figure 3:**
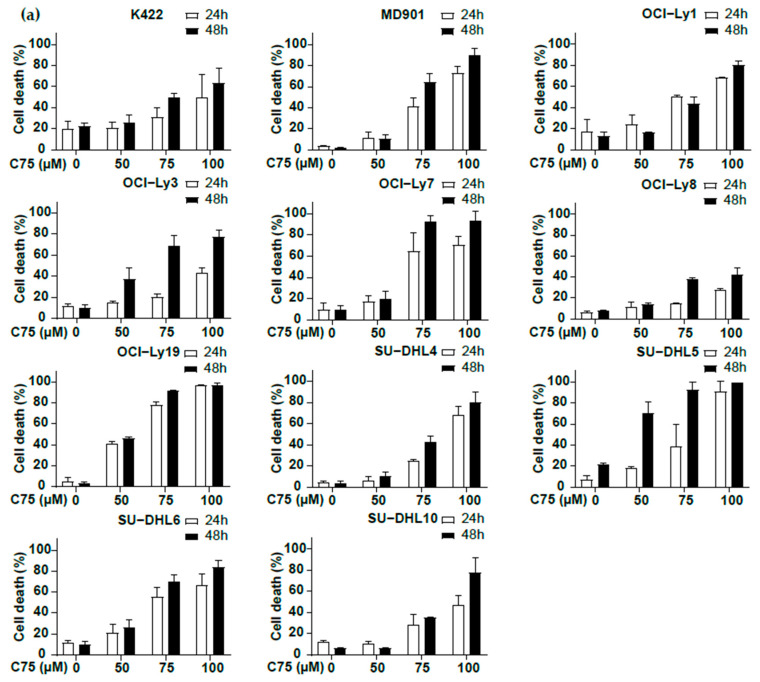

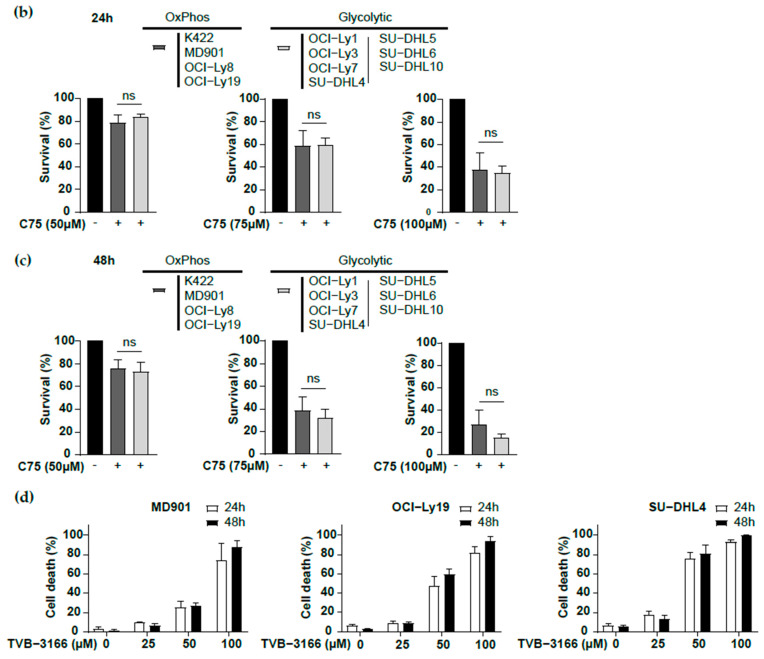
Inhibition of de novo fatty acid synthesis induces DLBCL cell death both in OxPhos and glycolytic DLBCL cell lines. (**a**) Inhibition of de novo fatty acid synthesis by the FASN inhibitor C75 induces cell death in DLBCL cell lines. The indicated DLBCL cell lines were treated with C75 at 50, 75, and 100 µM for 24 h and 48 h and monitored for cell death by trypan blue dye exclusion. Error bars represent means ± SD, at least *n* = 2 experiments. (**b**,**c**) DLBCL cell death induced by FA neosynthesis inhibition is independent of the OxPhos or glycolytic metabolic status. Cell survival relative to untreated cells set at 100% using C75 at 50 μM (left), 75 μM (middle), and 100 μM (right) for 24 h (**b**) and 48 h. (**c**) Control condition was set at 100%. Error bars represent means ± SEM, *p* values by unpaired Student’s *t*-test, ns: not significant. Dark grey columns represent OxPhos DLBCL cell lines and light grey columns represent glycolytic DLBCL cell lines. (**d**) Inhibition of de novo fatty acid synthesis by the reversible FASN inhibitor TVB-3166 induces cell death in DLBCL cell lines. MD901, OCI-Ly19, and SU-DHL4 were treated with TVB-3166 at 25, 50, and 100 µM for 24 h and 48 h and monitored for cell death by trypan blue dye exclusion. Error bars represent means ± SD (*n* = 3).

In contrast to normal cells, de novo lipogenesis is activated in cancer cells, providing selective proliferative and survival advantages [24,25]. Fatty acid synthase (FASN), a key multienzyme complex responsible for de novo lipogenesis, is upregulated in DLBCL patients [26,27,28]. Since the ascription of an oncogenic role for FASN, this enzyme has been considered a potential therapeutic target for cancer treatment [25,29]. Therefore, we undertook to evaluate how blockage of FASN activity impacts DLBCL survival. Treatment of the large panel of DLBCL cell lines with C75 led to dose-dependent (from 50 to 100 µM) DLBCL cell death (Figure 3a). Note that the cell killing induced by C75 is less efficient than 4-BrCA treatment (compare Figure 2a with Figure 3a). At the highest dose of C75 (100 µM) there was an average of 60% cell death, whereas the highest dose of 4-BCA (20 µM) resulted in only 20–30% of DLBCL cell survival after 24 h of treatment (Figure 3a,b). No significant difference in sensitivity to FASN inhibition was observed between OxPhos vs. glycolytic DLBCL cells (Figure 3b,c). Several reversible FASN inhibitors were recently developed by Sagimet Biosciences and have demonstrated antitumor activity in vitro and in vivo, along with some in phase II clinical trials [30]. Interestingly, treatment of DLBCL with TVB-3166 strongly impacted on DLBCL survival (Figure 3d) in a dose-dependent manner, thus reinforcing the beneficial impact of targeting FA neosynthesis. Collectively, our data show that both OxPhos and BCR/glycolytic DLBCL cell survival relies on FASN activity.

### 3.4. Combining Inhibition of FA Metabolism with Mitochondrial Stress Promotes DLBCL Cell Death

**Figure 4:**
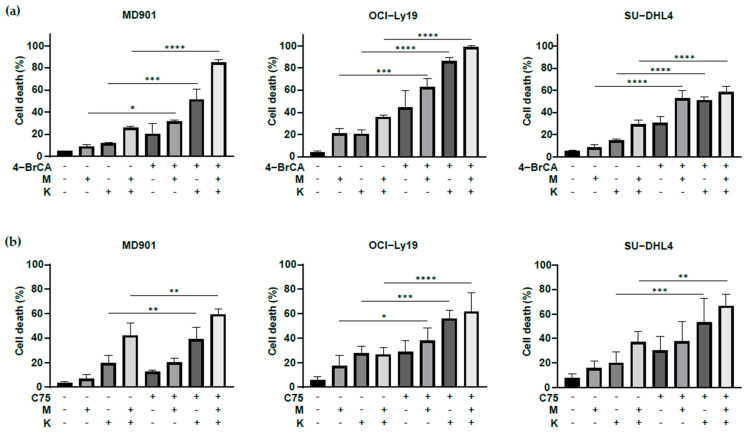
Targeting FA and mitochondrial metabolism strongly enhances DLBCL cell death irrespective of their OxPhos or glycolytic status. (**a**) MD901, OCI-Ly19, and SU-DHL4 DLBCL cell lines treated with 4-BrCA (15 μM) with the indicated mitochondrial metabolism inhibitors (M: metformin, 2.5 mM, K: L-asparaginase, 2 IU/mL) for 24 h and monitored for cell death by trypan blue dye exclusion. Error bars represent means ± SD, at least *n* = 2 experiments, *p* values by one-way ANOVA test, * *p* < 0.05; *** *p* < 0.001; **** *p* < 0.0001. M: metformin, K: L-asparaginase; (**b**) MD901, OCI-Ly19, and SU-DHL4 DLBCL cell lines treated with C75 with the indicated mitochondrial (M: metformin, 2.5 mM, K: L-asparaginase, 2 IU/mL) inhibitors for 24 h and monitored for cell death by trypan blue dye exclusion. C75: 50, 50 and 75 μM for MD901, OCI-Ly19, and SU-DHL4 DLBCL, respectively. Error bars represent means ± SD, at least *n* = 3 experiments, *p* values by one-way ANOVA test, * *p* < 0.05; ** *p* < 0.01; *** *p* < 0.001; **** *p* < 0.0001. M: metformin, K: Kidrolase (L-asparaginase).

Metformin is a widely prescribed anti-hyperglycemic biguanide drug for the treatment of type II diabetics, specifically inhibiting complex I of the mitochondrial electron transport chain [31]. L-asparaginase (^®^ Kidrolase) catalyzes the hydrolysis of asparagine and glutamine into aspartate and glutamate, respectively, accompanied by mitochondrial dysfunction [32,33,34,35]. Interestingly, combining L-asparaginase with either the FAO inhibitor 4-BrCA (Figure 4a) or the FASN inhibitor C75 (Figure 4b) significantly promoted DLBCL cell death. Similarly, the combination of FA metabolism inhibitors with metformin significantly reduced DLBCL cell survival (Figure 4a,b). Interestingly, the combination of metformin and L-asparaginase synergizes with lipid metabolism targeting to induce cell death in OxPhos and glycolytic DLBCL cell lines (Figure 4a,b, M + K).

Collectively, our findings demonstrate that targeting multiple metabolic pathways (lipids, mitochondria, glutamine) impacts more strongly on DLBCL cell survival.

### 3.5. Combination of FASN and mTORC1 Inhibition Promotes DLBCL Cell Death

**Figure 5:**
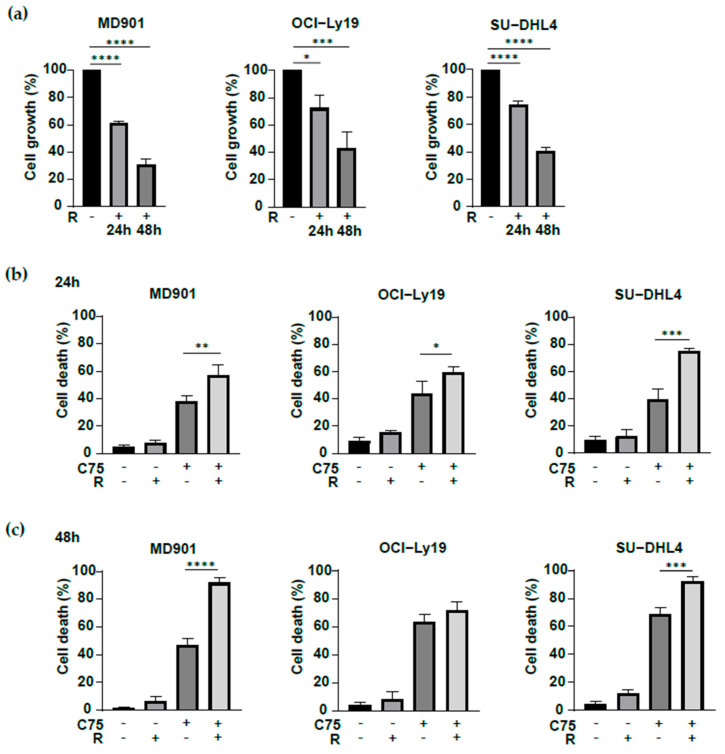
Combination of FASN and mTORC1 inhibition promotes DLBCL cell death. (**a**) mTORC1 inhibition slows down DLBCL cell growth. MD901, OCI-Ly19, and SU-DHL4 DLBCL cell lines were treated with rapamycin (20 nM) for the indicated time periods. Cell growth relative to untreated cells. Error bars represent means ± SD (*n* = 3), *p* values by unpaired Student’s *t*-test, * *p* < 0.05; *** *p* < 0.001; **** *p* < 0.0001. R: rapamycin. (**b**,**c**) Combination of FASN and mTOR inhibition potentiates OxPhos and glycolytic DLBCL cell death. MD901, OCI-Ly19, and SU-DHL4 DLBCL cell lines treated with the indicated antimetabolic inhibitors for 24 h (**b**) and 48 h (**c**) and monitored for cell death by trypan blue dye exclusion. R: rapamycin, 20 nM. C75: 75, 60, and 80 μM for MD901, OCI-Ly19, and SU-DHL4 DLBCL, respectively. Error bars represent means ± SD (*n* = 3), *p* values by unpaired Student’s *t*-test, * *p* < 0.05; ** *p* < 0.01; *** *p* < 0.001; **** *p* < 0.0001.

The mTOR oncogenic signaling pathway plays a central role in lipogenesis [36,37,38,39] and has been reported to play an important role in DLBCL cell survival [40,41]. Therefore, we have undertaken to evaluate the impact of the mTOR inhibitor rapamycin either alone or in combination with the FA neosynthesis inhibition. mTORC1 inhibition has a strong cytostatic effect (Figure 5a) but does not induce DLBCL cell death (Figure 5b,c). Remarkably, combining rapamycin with FASN inhibition promotes DLBCL cell death (Figure 5b,c), therefore indicating that combining inhibition of the mTOR oncogenic pathway and FA metabolism has a broader metabolic impact on DLBCL cells, thereby impacting more strongly of their survival (both OxPhos and glycolytic, ABC and GCB).

## 4. Discussion

Reprogramming of energy metabolism is a well-established hallmark of cancer cells [15]. Recently, FA metabolism has emerged as a central player in altered energetics in cancers affecting cancer cell survival [17,18,42]. FAs are described as an energy source through the process of FAO in the mitochondria. The upregulation of FA synthesis is a classically described metabolic alteration in cancer [24,25,43]. In the study presented here, we conducted the first comprehensive analysis of how inhibition of FA metabolism impacts DLBCL survival using a large panel of eleven DLBCL cell lines characterized by their metabolic status (OxPhos vs. glycolytic) and cell of origin (ABC vs. GCB). We uncovered that DLBCL cells are sensitive to drugs targeting either lipid oxidation for energy production, or the lipogenic enzyme FASN, in all subtypes. First, we have shown that FAO inhibition targeting the 3-ketoacyl-CoA thiolase, the enzyme of the last step of β-oxidation [23] induces massive DLBCL cell death in both the OxPhos and glycolytic metabolic subtypes. Second, targeting the lipogenic enzyme FASN using the C75 inhibitor leads to significant DLBCL cell death. Third, the small molecule TVB-3166 developed by Sagimet Biosciences also induces strong DLBCL cell death. Whether such observations are exclusive to DLBCL or can be extended to other types of hematological cancers would be worth further investigation.

FASN, one of the lipogenic enzymes, has been known for many years to be upregulated in several cancer types, including DLBCL [25,29]. Recently, FASN overexpression has been associated with the development of a more aggressive phenotype and poor DLBCL patient survival [26,28,44]. Still, the exact molecular mechanisms underlying dysregulation of FA metabolism in DLBCL cells and their relation to the inferior clinical outcome of patients have remained largely elusive, which is worth further investigation. Few previous studies using a very low number of DLBCL cell lines suggested that the ABC DLBCL subset may be more sensitive to FASN inhibition, although with seemingly contradictory results [27,28,44,45]. Being the first to use a large panel of GCB DLBCL cell lines, our study clearly demonstrates that DLBCL cells of the GCB subtype are sensitive to FASN inhibition.

Our study shows that DLBCL cells are highly sensitive to inhibition of FAO and de novo FA synthesis. Nonetheless, the associated molecular mechanisms remain unclear. Interestingly, we have observed a cooperative effect of targeting FASN and mTORC1 activity to induce DLBCL cell death. The mTORC1 oncogenic pathway promotes lipogenesis [36,38]. A link between FASN and aberrant protein translation involving mTORC1 signaling has been reported in DLBCL cells [27,46]. In addition, it has been reported that TVB-3166 stimulated cancer cell apoptosis by modulating the mTOR pathway along with altering the lipid profiles [30]. Whether the cooperativity between FASN and mTORC1 is linked to altered control of protein translation or other molecular mechanisms in DLBCL remains an open question.

Cancer cells are very adaptable and exhibit an amazing metabolic plasticity in the most adverse conditions [42]. A metabolomics analysis of the large panel of DLBCL cell lines as well as a direct measure of their mitochondrial oxidative metabolism would be informative. Many clinical trials using individual metabolic inhibitors have failed to improve the outcome of DLBCL patients [47,48,49]. This may illustrate the ability of cancer cells to find a metabolic path to reprogram and bypass the targeted metabolic pathway. In this study, we demonstrated that combining inhibition of FA metabolism and induction of mitochondrial stress (metformin and/or L-asparaginase) triggers massive DLBCL cell death. The beneficial combinatory effect was seen in OxPhos and BCR/glycolytic DLBCL cell lines, thus highlighting the benefit of inducing a wide spectrum of metabolic disturbances to promote optimal DLBCL cell death.

In summary, by using for the first time a large panel of DLBCL cell lines, we established that drugs targeting FA metabolism induce massive DLBCL cell death independently of their metabolic status. Further, we show that simultaneous targeting of FA metabolism and other metabolic-linked survival pathways, including glutamine and mitochondrial metabolism, improves DLBCL cell killing. Our data are of great functional importance because they constitute a significant advance in the understanding of metabolic vulnerabilities of DLBCL cells and provide a strong rationale for the use of compounds targeting FA metabolism as adjuvant therapy to enhance current treatment protocols in R/R DLBCL patients. Numerous drugs and small-molecule inhibitors with the goal to inhibit cancer cell lipid metabolism are under development. As this area of research is moving at a rapid pace, there is hope for lipid-targeted therapeutic strategies to find their path in the clinic.

## Data Availability

Data are all contained within the article.
